# Tree Nut consumption is associated with better adiposity measures and cardiovascular and metabolic syndrome health risk factors in U.S. Adults: NHANES 2005–2010

**DOI:** 10.1186/s12937-015-0052-x

**Published:** 2015-06-28

**Authors:** Carol E. O’Neil, Victor L. Fulgoni, Theresa A. Nicklas

**Affiliations:** 1 Louisiana State University Agricultural Center, 261 Knapp Hall, 110 LSU Union Square, Baton Rouge, LA 70803 USA; 2 Nutrition Impact, LLC, 9725 D Drive North, Battle Creek, MI 49014 USA; 3 Department of Pediatrics, USDA/ARS Children’s Nutrition Research Center, Baylor College of Medicine, 1100 Bates Street, Houston, TX 77030 USA

**Keywords:** Tree Nuts, NHANES, Adults, Metabolic Syndrome, Cardiovascular Risk Factors

## Abstract

**Introduction:**

Previous research has shown inconsistencies in the association of tree nut consumption with risk factors for cardiovascular disease (CVD) and metabolic syndrome (MetS).

**Objective:**

To determine the association of tree nut consumption with risk factors for CVD and for MetS in adults.

**Methods:**

NHANES 2005–2010 data were used to examine the associations of tree nut consumption with health risks in adults 19+ years (*n* = 14,386; 51 % males). Tree nuts were: almonds, Brazil nuts, cashews, filberts [hazelnuts], macadamias, pecans, pine nuts, pistachios, and walnuts. Group definitions were non-consumers < ¼ ounce/day and consumers of ≥ ¼ ounce/day tree nuts using data from 24-h dietary recalls. Means and ANOVA (covariate adjusted) were determined using appropriate sample weights. Using logistic regression, odds ratios of being overweight (OW)/obese (OB) (body mass index [BMI] >25/<30 and ≥30, respectively) and having CVRF or MetS, were determined.

**Results:**

Tree nut consumption was associated with lower BMI (*p* = 0.004), waist circumference (WC) (*p* = 0.008), systolic blood pressure (BP) (*p* = 0.001), Homeostatic Model Assessment—Insulin Resistance (*p* = 0.043), and higher high density lipoprotein-cholesterol (*p* = 0.022), compared with no consumption, and a lower likelihood of OB (−25 %), OW/OB (−23 %), and elevated WC (−21 %).

**Conclusions:**

Tree nut consumption was associated with better weight status and some CVRF and MetS components.

## Introduction

Tree nuts have been part of the diet of humans since paleolithic times [1]. The nutrients found in tree nuts, including almonds, Brazil nuts, cashews, filberts [hazelnuts], macadamias, pecans, pine nuts, pistachios, and walnuts vary by species, but in general, they provide energy, vegetable protein, heart-healthy oils, including monounsaturated fatty acids (MUFA) and polyunsaturated fatty acids (PUFA), dietary fiber, calcium, potassium, folate, magnesium, selenium, and vitamin E. Tree nuts are also low in sodium and have no cholesterol [2]. Coupled with this positive nutrient profile, tree nuts also provide phenols, phytosterols, flavonoids, resveratrol, and other bioactive compounds [2, 3], which when coupled with vitamin E and selenium, serve as anti-oxidants, which may reduce the risk of cardiovascular risk factors (CVRF) [4–7] and cardiovascular disease [8, 9].

Recently, tree nut consumption was shown to have a significant inverse association with all-cause mortality and with death due to heart disease [8, 10]. The study by Bao et al., [8] showed that the frequency of tree nut consumption was associated with decreased risk for heart and cardiovascular disease, with the number of deaths lowest in those consuming tree nuts five or more times a week. That study failed to show a significant association with frequency of tree nut consumption and death due to stroke or type 2 diabetes.

Most previous feeding studies have shown that consumption of tree nuts has been associated with healthier levels of CVRF, including total cholesterol [7, 11–13], low-density lipoprotein cholesterol (LDL-C) [7, 11–15], high-density lipoprotein cholesterol (HDL-C) [7, 14], triglycerides [7], apolipoprotein A [13, 16], and apolipoprotein B [11]; markers of oxidative stress [17, 18] or inflammatory markers [19]; endothelial dysfunction [7, 20, 21]; insulin resistance [22, 23]; hyperglycemia [15]; and hemoglobin A1c [15]. However, other studies have shown no significant effects on total cholesterol [24, 25], LDL-C [24, 25], HDL-C [24], triglycerides [24, 25], C-reactive protein (CRP) [16, 24], fasting blood sugar [25], insulin resistance [21], hemoglobinA1c [21], and serum fructosamine [24].

Cross-sectional studies of adults that have examined the association between tree nut consumption and CVRF have also shown conflicting results. Tree nut consumers have been shown to have lower values for or decreased risk of higher body mass index (BMI) [5], obesity [4], elevated waist circumference (WC) [5], low HDL-C [5, 6], CRP [6], lower systolic (SBP) or diastolic blood pressure (DBP) [5, 6, 26], elevated fasting glucose [5], hemoglobin A1c [6], insulin [6], and a lower prevalence of metabolic syndrome (MetS) [5]. These studies all looked at multiple CVRF and findings were inconsistent since they also showed that tree nut consumption was not associated with decreased values or decreased risk of higher weight [6], elevated WC [6], components of dyslipidemia [4], hypertension [4], elevated fasting glucose [4, 6], or MetS [4, 6].

Reasons for these conflicting results in feeding and epidemiologic studies are not clear, but may include the populations studied, the type and amount of tree nut consumed, the length of the feeding trial, and, in the epidemiologic studies the method of classifying consumers into groups. Disparities among these studies indicate the need for further studies. There have been no recent studies using nationally representative data that has looked specifically at tree nut consumption and CVRF. The purpose of this study was to examine this association, using current data from participants of the National Health and Nutrition Examination Survey (NHANES) 2005–2010.

## Subjects and methods

### Study population and analytic sample

Data from the NHANES 2005–2006, 2007–2008, and 2009–2010 datasets were used to evaluate tree nut or tree nut butter (these components were considered together and are referred to as tree nuts below) consumption in the US population. Data from adults 19+ years of age (y) (*N* = 14,386) participating in the NHANES were combined to increase sample size [27, 28]. Analyses included only individuals with complete and reliable dietary recalls as determined using the National Center for Health Statistics staff. Females who were pregnant or lactating were excluded from the study. In compliance with federal law, the NHANES use defined strict protocols to ensure confidentiality and protect participants’ identity. As this study used secondary data, stripped of individual identifiers, it did not require institutional review [29].

Demographic information, including age, gender, race-ethnicity, poverty index ratio (PIR), physical activity levels, and smoking status, used for covariates in the statistical analyses outlined below, was determined via interview [30]. Alcohol was also used as a covariate and was determined using the 24-h dietary recalls described below.

### Dietary analyses

Dietary intake was determined using two multiple pass 24-h dietary recalls [31, 32]. The first recall was in-person in the Mobile Examination Center [33] and the second was conducted 3–10 days later via telephone [34]. The US Environmental Protection Agency Food Commodity Intake Database (FCID) commodity codes [35] were used to identify ingredients of survey foods that included tree nuts: almonds, Brazil nuts, cashews, filberts [hazelnuts], macadamias, pecans, pine nuts, pistachios, and walnuts.

The gram amount of tree nuts consumed by NHANES 2005–2010 respondents was determined by applying the FDIC tree nut composition data to the respondent’s 24-h recall dietary interview data. Tree nut intakes were aggregated over the entire day. Usual intake (UI) was determined using the National Cancer Institute method with survey day (one or two) and a weekend day flag (Friday/Saturday/Sunday versus others) as covariates [36]. Tree nut or all nut consumers were defined by a UI of at least ¼ ounce (7.0875 grams) per day [5].

### Anthropometric and physiologic measures

Height, weight, and WC were obtained according to NHANES protocols [37]. Body Mass Index (BMI) was calculated as body weight (kilogram) divided by height (meters) squared [38]. Systolic blood pressure and DBP were determined using the standard NHANES protocol [39]. High density lipoprotein-cholesterol were determined on non-fasted individuals [40] while LDL-C [41], triglycerides [41], blood glucose [42], and insulin [42] were determined on only fasted subjects; thus, not all individuals may have values for all tests.

Overweight/obesity and high WC were determined using the National Heart Lung and Blood Institute Clinical Guidelines [38]. Overweight was defined as a BMI >25 and ≤29.9; obesity was defined as a BMI ≥ 30 kg/m^2^. High WC was defined as >102 cm for males and >88 cm in females. The Homeostatic Model of Assessment-Insulin Resistance (HOMA-IR), used to evaluate insulin resistance, was calculated as: fasting serum insulin/fasting plasma glucose [43]. Metabolic syndrome (MetS) was defined using the National Heart Lung and Blood Institute Adult Treatment Panel III criteria [44]: having 3 or more of the following risk factors: abdominal obesity, WC > 102 cm (males), >88 cm (females); hypertension, SBP ≥130 mmHg or DBP ≥85 mmHg or taking anti-hypertensive medications; HDL-cholesterol, <40 mg/dL (males), <50 mg/dL (females); high triglycerides, ≥150 mg/dL or taking anti-hyperlipidemic medications; high fasting glucose, ≥110 mg/dL or taking insulin or other hypoglycemic agents. An elevated LDL-C was defined as ≥100 mg/dL.

### Statistical analyses

Sample-weighted data were used in all statistical analyses; and, all analyses were performed using SUDAAN Release11.0 (Research Triangle Institute, Research Triangle Park, NC) to adjust the variance for the complex sample design. For the 6-years 2005–2010, a 6-year weight variable was created by assigning ${\scriptscriptstyle \frac{1}{3}}$ of the 2 year weight for 2005–2006, 2007–2008, and 2009–2010 [27, 28]. A 6-year MEC-examined sample weight was used in analyses of intake, body measurements, blood pressure, and laboratory data, except a 6-year fasted sample weight was used in analyses of LDL-C, triglycerides, plasma glucose, insulin, and MetS.

The sample-weighted percentages (and standard error of the percentages) of the adults in tree nut consumers were calculated using PROC DESCRIPT of SUDAAN. Least-square means (and the standard errors of the least-square means) were calculated using PROC REGRESS of SUDAAN. The adjusted prevalence of a risk factor was determined by calculating the least-square mean of a dichotomous variable using PROC REGRESS, and odds ratios were calculated using PROC LOGISTIC of SUDAAN.

Covariates for least-square mean values and odds ratios of weight/adiposity related variables were, gender, age (years), race-ethnicity, poverty index ratio, physical activity level, smoking status and alcohol intake. The least-square mean values and odds ratios of BP, blood lipids, fasting glucose, and insulin were adjusted for BMI (kg/m^2^) as well. A p value of <0.05 was considered significant.

## Results

### Nut consumption

Tree nut consumers (*n* = 755; 50.2 % female; mean age 51.18 years ± 0.42 SE) constituted approximately 6.8 % of the population. Details of the demographics of this population have been published previously [45]. Mean UI of tree nut consumers was 44.3 ± 1.6 g/d; whereas, *per captia* UI was 3.3 ± 0.1 g/d.

### Weight/adiposity measures/blood pressure

Table 1 shows tree nut consumers had better weight/adiposity parameters than non- consumers. BMI (27.9 ± 0.3 v 28.7 ± 0.1 kg/m^2^; *p* = 0.004), and WC (95.8 ± 0.7 v 98.1 ± 0.3 cm; *p* = 0.008) were all significantly lower in tree nut consumers. Systolic blood pressure was lower in tree nut consumers (119.5 ± 0.8 v 122.1 ± 0.2 mm Hg; p = 0.001).

**Table 1:** The association of consuming tree nuts with weight/adiposity and blood pressure measurements in adults participating in the 2005–2010 National Health and Nutrition Examination Survey

Variable	Number	Tree Nut Consumers LS Mean ± SE	Non-Consumers LS Mean ± SE	*p*
Weight (kg)^a^	14,229	80.7 ± 0.9	82.2 ± 0.3	0.102
BMI (kg/m^2^)^a^	14,204	27.9 ± 0.3*	28.7 ± 0.1*	0.004
WC (cm)^a^	13,838	96.1 ± 0.7*	98.0 ± 0.3*	0.008
Systolic BP (mm Hg)^b^	13,918	119.5 ± 0.8*	122.1 ± 0.2*	0.001
Diastolic BP (mm Hg)^b^	13,851	71.8 ± 0.8	70.6 ± 0.3	0.221

Abbreviations: BMI = Body mass index, WC = Waist circumference, BP = Blood pressure

^a^Covariates: Gender, Ethnicity, Age, Poverty Index Ratio, Physical Activity Level (sedentary, moderate, active), Current Smoker Status, and Alcohol

^b^Covariates: Gender, Ethnicity, Age, Poverty Index Ratio, Physical Activity Level (sedentary, moderate, active), Current Smoker Status, Alcohol, and BMI

*Significantly Different

### Physiologic measures

Table 2 shows that tree nut consumers had higher HDL-C levels (54.4 ± 0.6 v 52.9 ± 0.3 mg/dL; p = 0.022) and lower HOMA-IR values (3.0 ± 0.1 v 3.3 ± 0.1; p = 0.043) than non-consumers. Odds ratio analyses (Table 3) showed that tree nut consumers had a 25 % lower likelihood of obesity (OR = 0.75; 95 % confidence interval [CI] 0.60-0.95), a 23 % lower likelihood of overweight or obesity (0.77; 0.62-0.95), and a 21 % lower likelihood of an elevated WC (0.79; 0.64-0.99) than non-consumers.

**Table 2:** The association of consuming tree nuts with physiologic measures in adults participating in the 2005–2010 National Health and Nutrition Examination Survey

Variable	Number	Tree Nut Consumers LS Mean ± SE	Non-Tree Nut Consumers LS Mean ± SE	*p*
LDL-C (mg/dL)^a^	6480	115.5 ± 2.4	115.8 ± 0.6	0.902
HDL-C (mg/dL)^a^	13,666	54.4 ± 0.6*	52.9 ± 0.3*	0.022
Triglycerides (mg/dL)^b^	6621	127.4 ± 6.5	134.1 ± 1.9	0.344
Glucose (mg/dL)^c^	6662	102.1 ± 1.2	104.3 ± 0.4	0.061
Insulin (uU/mL)	6581	11.3 ± 0.5	12.1 ± 0.2	0.125
HOMA-IR	6568	3.0 ± 0.1*	3.3 ± 0.1*	0.043
CRP (mg/dL)^d^	13,709	0.4 ± 0.04	0.4 ± 0.01	0.276

Abbreviations: BMI = body mass index, WC = waist circumference, BP = blood pressure, LDL-C = low-density lipoprotein cholesterol, HDL-C = high-density lipoprotein cholesterol, HOMA-IR = Homeostatic Model of Assessment - Insulin Resistance, CRP = C-reactive protein

Covariates: Gender, Ethnicity, Age, Poverty Index Ratio, Physical Activity Level (sedentary, moderate, active), Current Smoker Status, Alcohol, and BMI

^a^To convert mg/dL to mmol/L divide by 38.67

^b^To convert mg/dL to mmol/L divide by 38.67

^c^To convert mg/dL to mmol/L multiply by 0.055

^d^To convert mg/dL to mmol/L multiply by 9.524

*Significantly Different

**Table 3:** Risk of overweight and obesity and cardiovascular and metabolic syndrome risk factors in adult consumers and Non-consumers of tree nuts participating in the 2005–2010 National Health and Nutrition Examination Survey

Variable	OR	LCL	UCL
Overweight^a,b^	1.02	0.67	1.24
Obese^a,b^	0.75*	0.60	0.95
Overweight or obese^a,b^	0.77*	0.62	0.95
WC elevated^a,c^	0.79*	0.64	0.99
Elevated systolic BP^d^	0.91	0.71	1.17
Elevated diastolic BP^d^	1.03	0.80	1.33
LDL-C elevated^d,e^	0.80	0.57	1.14
HDL-C reduced^d,e^	0.85*	0.67	1.07
Triglycerides elevated^d,e^	0.81	0.58	1.13
Glucose elevated^d,e^	0.81	0.59	1.11
Insulin elevated (>85th Pctl)^d^	0.82	0.54	1.26
Metabolic syndrome^a,f^	0.74	0.52	1.05

Abbreviations: WC = Waist circumference, BP = Blood pressure, LDL-C = Low-density lipoprotein cholesterol, HDL-C = High-density lipoprotein cholesterol

For Tree Nut Consumers, the reference group was no tree nut consumption

^a^Covariates: Gender, Ethnicity, Age, Socioeconomic Status (PIR 0–1.25, 1.25-3.4,  ≥ 3.25), Physical Activity Level (sedentary, moderate, active), Current Smoker Status, and Alcohol

^b^Oveweight was defined as a BMI 25–29.9; obese was defined as a BMI ≥ 30; overweight or obese was defined as a BMI ≥25

^c^Elevated WC was defined as >102 cm (males), >88 cm (females)

^d^Covariates: Gender, Ethnicity, Age, Socioeconomic Status (PIR 0–1.25, 1.25-3., ≥ 3.25), Physical Activity Level (sedentary, moderate, active), Current Smoker Status, Alcohol, and BMI

^e^Reduced HDL-cholesterol was defined as <40 mg/dL (males), <50 mg/dL (females); high triglycerides, ≥150 mg/dL or taking anti-hyperlipidemic medications; high fasting glucose, ≥110 mg/dL or taking insulin or other hypoglycemic agents. Elevated LDL-C ≥100 mg/dL

^f^Metabolic syndrome was defined using the National Heart Lung and Blood Institute Adult Treatment Panel III criteria; that is having 3 or more of the following risk factors: abdominal obesity, WC > 102 cm (males), >88 cm (females); hypertension, SBP ≥130 mmHg or DBP ≥85 mmHg or taking anti-hypertensive medications; HDL-cholesterol, <40 mg/dL (males), <50 mg/dL (females); high triglycerides, ≥150 mg/dL or taking anti-hyperlipidemic medications; high fasting glucose, ≥110 mg/dL or taking insulin or other hypoglycemic agents

*p < 0.05

## Discussion

This study showed that those consuming tree nuts had better weight/adiposity measures and a lower risk of obesity, overweight/obesity, and elevated WC than non-consumers. Tree nut consumers also had lower SBP and higher levels of HDL-C. The association of tree nut consumption and weight and cardiovascular risk factors using NHANES data has not been examined since the 1999–2004 data sets were published. An advantage to using data sets published after 2001–2002 cycle is that two 24-h dietary recalls are available from participants. Thus, since UI can be calculated [36], concerns about using a single dietary recall in data analysis should be assuaged.

Always of interest is to compare secular trends in consumption of healthful foods, like tree nuts. However, it is difficult to compare the percentage of individuals consuming tree nuts and the amount consumed by individuals in three previous NHANES studies [5, 6, 46], since the earlier studies used a single 24 h dietary recall and this study used UI. Further, this study used the FCID commodity codes [35] to determine intake, as opposed to the food codes found in Food and Nutrient Database for Dietary Studies [47] which are often used [5, 6]. The advantage is that the FDIC database provides estimates of food consumption, in terms of ingredients or as the food “as eaten.” In this study, approximately 6.8 % of the study population consumed tree nuts; although this seems low, the weighted number actually represented over 12,440,000 million individuals—a significant number. Those consuming tree nuts consumed an average of 44.3 g which is higher than the ½ ounce (~14 g) that is considered an ounce equivalent of a protein food by MyPlate and higher than the US Food and Drug Administration’s reference amount customarily consumed of 30 g for all types of nuts and mixtures except butters [48]. It is similar to the 1½ ounces (42.5 g) recommended in the qualified health claim for tree nuts and heart disease [49].

In this study tree nut consumers had lower mean weight, BMI, and WC than non-consumers. There was also a lower risk of obesity/overweight, obesity, and elevated WC. Although tree nuts are an energy dense food, an inverse association between tree nut consumption and weight parameters or weight gain has been shown previously in cross-sectional studies [4, 5], prospective long-term cohort studies [50, 51], and feeding studies [52, 53]. A recent meta-analysis of controlled clinical trials looking at nut consumption and weight has also shown that diets “enriched with nuts” did not increase weight or measures of adiposity [54].

The biological plausibility for these findings has been offered previously [54, 55]. Due to their high vegetable protein, dietary fiber, MUFA, and PUFA content, tree nuts are a satiating food and following consumption, appetite and consequently intake may be suppressed at subsequent eating occasions. Nuts must be chewed so that the particles are small enough to be swallowed; mastication may modify appetite. Further, the energy in nuts may be inefficiently absorbed. Finally, Atwater factors, when applied to almonds [56] and pistachios [57] resulted in a 32 % and 5 % overestimation, respectively, of their measured energy content. Obesity also contributes to the major causes of morbidity and mortality in the US; thus, any dietary changes that can lower the risk of obesity should be encouraged.

This study and both earlier studies [5, 6] of NHANES participants have shown lower SBP in tree nut consumers than in non-consumers. With relatively high levels of unsaturated fatty acids, calcium (almonds), potassium, magnesium, and dietary fiber, coupled with low levels of sodium [2], tree nuts would appear to be a food associated with low blood pressure and they are encouraged in the DASH diet [58]. However, studies have shown that the effect of tree nut consumption on blood pressure is inconsistent. The cross-sectional PREDIMED study did not show an effect of tree nut consumption on hypertension [4]. Data from prospective cohorts are limited. Participants in the Physicians’ Health Study I [59] reported a lower incidence of hypertension in lean men only; however, in the SUN study there was not relationship between nut consumption and incident hypertension [60]. A review of 19 clinical trials looking at blood pressure and nut consumption showed inconsistent results, with 13 studies showing no changes in blood pressure, one showing an increase in blood pressure, and the remaining five studies showing a reduction [61].

Consistent with other cross-sectional studies [5, 6], this study showed higher HDL-C levels in tree nut consumers than in non-consumers. Cross-sectional studies are hypothesis generating; thus, these findings led, in part, to clinical trials (hypothesis testing) that examined diets containing nuts versus those not containing nuts. These clinical trials have shown inconsistent results with regard to HDL-C levels. For example, Tappsell, et al., [62] showed an increase in HDL-C levels in individuals after 6 months of consuming a diet containing walnuts as compared to those consuming a control diet; whereas, Sabaté, et al., [63] showed that HDL-C levels were lower in those consuming 20 % of energy from walnuts, as compared with those consuming the control diet. Both of these studies were conducted in specific groups, the first in diabetics and the second in men only. To help reconcile these findings, a recent pooled analysis of primary data from 25 tree nut consumption trials with a total of 583 participants failed to show a significant difference in mean HDL-C levels between tree nut consumers and non-consumers [64]. Reasons for the differences between the results of cross-sectional studies, such as NHANES, and clinical trials are not clear but may reflect the population used, the length of the study, the amount of specific tree nuts consumed, and the assignment to consumption groups in cross-sectional studies. Overall, the relationship between lipid levels and tree nut consumption has been inconsistent, but overall, the association is positive [63]. This is likely due to the low saturated fatty acids, high MUFA, PUFA, and phytochemical content of most tree nuts.

Metabolic Syndrome is characterized by dyslipidemia, hypertension, abdominal obesity, insulin resistance, and hyperglycemia; it is a major risk factor for cardiovascular disease and type 2 diabetes [65]. It has previously been shown that tree nut consumers have a lower prevalence of MetS [5], but a previous cross-sectional study that looked only at out-of-hand tree nut and peanut consumption [6] failed to show that nut consumption was associated with a reduced risk of MetS. One reason may be that that study failed to show a difference in several of the risk factors for MetS, including elevated WC, triglycerides, and fasting glucose. Since this study also failed to show a reduced risk of MetS in tree nut consumers, additional studies are warranted.

Differences among results from the cross-sectional, cohort, and feeding studies seen in the CVRF examined may be the result of different tree nuts used in individual feeding studies, which may reflect the different nutrient profile of individual nut species; how consumption was determined, e.g., amount or frequency; the inflammatory markers studied; the characteristics of the population tested, including gender or whether participants were healthy or had been diagnosed with obesity, MetS, hyperlipidemics, or diabetes; or the length of the study.

### Strengths and limitations

The strengths of this study included a large, nationally representative population and use of UI in the analyses. The limitations of the study are that results from any cross-sectional epidemiologic study cannot be used to determine cause and effect. Also since these data are based on self-reported intake, it must be considered whether tree nuts are reported differently than other foods. If self-reported intake of nuts is different from other foods it may make it more likely that consumers and non-consumers were misclassified.

### Conclusions and implications

The prevalence of tree nut consumers was low; however, consumption was associated with a better weight/adiposity and CVRF profile than seen in non-consumers. Health professionals, especially registered dietitians and other health educators should provide diet counseling and nutrition education programs that increase awareness of the health benefits of tree nut consumption. Tree nuts should be consumed as part of an overall healthful meal pattern. Because of the conflicting results produced when studying the health benefits of tree nuts, future research should include more longitudinal studies and intervention trials examining these potential benefits.

## Abbreviations

BMI: Body mass index
BP: Blood pressure
CPR: C-Reactive protein
CVRF: Cardiovascular risk factors
DBP: Diastolic blood pressure
FCID: Food commodity intake database
HDL-C: High density lipoprotein-cholesterol
HOMA-IR: Homeostatic model of assessment—insulin resistance
LDL-C: Low density lipoprotein-cholesterol
MetS: Metabolic syndrome
MUFA: Monounsaturated fatty acids
NHANES: National health and nutrition examination survey
OB: Obesity
OW: Overweight
PUFA: Polyunsaturated fatty acids
SBP: Systolic blood pressure
UI: Usual intake
WC: Waist circumference

## References

[1] Salas-Salvadó J, Casas-Agustench P, Salas-Huetos A (2011). Cultural and historical aspects of Mediterranean nuts with emphasis on their attributed healthy and nutritional properties. Nutr Metab Cardiovasc Dis.

[2] U.S. Department of Agriculture, Agricultural Research Service. 2013. USDA National Nutrient Database for Standard Reference, Release 26. Nutrient Data Laboratory Home Page. [http://www.ars.usda.gov/ba/bhnrc/ndl]

[3] Maguire LS, O'Sullivan SM, Galvin K, O'Connor TP, O'Brien NM (2004). Fatty acid profile, tocopherol, squalene and phytosterol content of walnuts, almonds, peanuts, hazelnuts and the macadamia nut. Int J Food Sci Nutr.

[4] Ibarrola-Jurado N, Bulló M, Guasch-Ferré M, Ros E, Martínez-González MA, Corella D, Fiol M, Wärnberg J, Estruch R, Román P, Arós F, Vinyoles E, Serra-Majem L, Pintó X, Covas MI, Basora J, Salas-Salvadó J (2013). PREDIMED Study Investigators. Cross-sectional assessment of nut consumption and obesity, metabolic syndrome and other cardiometabolic risk factors: the PREDIMED study. PLoS One.

[5] O’Neil CE, Keast DR, Nicklas TA, Fulgoni VL (2011). Nut consumption is associated with decreased health risk factors for cardiovascular disease and metabolic syndrome in U.S. adults: NHANES 1999–2004. J Am Coll Nutr.

[6] O'Neil CE, Keast DR, Nicklas TA, Fulgoni VL (2012). Out-of-hand nut consumption is associated with improved nutrient intake and health risk markers in US children and adults: National Health and Nutrition Examination Survey 1999–2004. Nutr Res.

[7] Orem A, Yucesan FB, Orem C, Akcan B, Kural BV, Alasalvar C, Shahidi F (2013). Hazelnut-enriched diet improves cardiovascular risk biomarkers beyond a lipid-lowering effect in hypercholesterolemic subjects. J Clin Lipidol.

[8] Bao Y, Han J, Hu FB, Giovannucci EL, Stampfer MJ, Willett WC, Fuchs CS (2013). Association of nut consumption with total and cause specific mortality. N Engl J Med.

[9] Stradling C, Hamid M, Fisher K, Taheri S, Thomas GN (2013). A review of dietary influences on cardiovascular health: part 1: the role of dietary nutrients. Cardiovasc Hematol Disord Drug Targets.

[10] Guasch-Ferré M, Bulló M, Martínez-González MÁ, Ros E, Corella D, Estruch R, Fitó M, Arós F, Wärnberg J, Fiol M, Lapetra J, Vinyoles E, Lamuela-Raventós RM, Serra-Majem L, Pintó X, Ruiz-Gutiérrez V, Basora J, Salas-Salvadó J (2013). PREDIMED study group. Frequency of nut consumption and mortality risk in the PREDIMED nutrition intervention trial. BMC Med.

[11] Wu L, Piotrowski K, Rau T, Waldmann E, Broedl UC, Demmelmair H, Koletzko B, Stark RG, Nagel JM, Mantzoros CS, Parhofer KG (2014). Walnut-enriched diet reduces fasting non-HDL cholesterol and apolipoprotein B in healthy Caucasian subjects: A randomized controlled cross-over clinical trial. Metabolism.

[12] Ros E, Núñez I, Pérez-Heras A, Serra M, Serra M, Gilabert R, Casals E, Deulofeu R (2004). A walnut diet improves endothelial function in hypercholesterolemic subjects: a randomized crossover trial. Circulation.

[13] Zambón D, Sabaté J, Muñoz S, Campero B, Casals E, Merlos M, Laguna JC, Ros E (2004). Substituting walnuts for monounsaturated fat improves the serum lipid profile of hypercholesterolemic men and women. A randomized crossover trial. Ann Intern Med.

[14] Colpo E, Vilanova CD, Brenner Reetz LG, Medeiros Frescura Duarte MM, Farias IL, Irineu Muller E, Muller AL, Moraes Flores EM, Wagner R, da Rocha JB (2013). A single consumption of high amounts of the Brazil nuts improves lipid profile of healthy volunteers. J Nutr Metab.

[15] Jenkins DJ, Kendall CW, Banach MS, Srichaikul K, Vidgen E, Mitchell S, Parker T, Nishi S, Bashyam B, de Souza R, Ireland C, Josse RG (2011). Nuts as a replacement for carbohydrates in the diabetic diet. Diabetes Care.

[16] Aronis KN, Vamvini MT, Chamberland JP, Sweeney LL, Brennan AM, Magkos F, Mantzoros CS (2012). Short-term walnut consumption increases circulating total adiponectin and apolipoprotein A concentrations, but does not affect markers of inflammation or vascular injury in obese humans with the metabolic syndrome: data from a double-blinded, randomized, placebo-controlled study. Metabolism.

[17] Jenkins DJ, Kendall CW, Josse AR, Salvatore S, Brighenti F, Augustin LS, Ellis PR, Vidgen E, Rao AV (2006). Almonds decrease postprandial glycemia, insulinemia, and oxidative damage in healthy individuals. J Nutr.

[18] Torabian S, Haddad E, Rajaram S, Banta J, Sabaté J (2009). Acute effect of nut consumption on plasma total polyphenols, antioxidant capacity and lipid peroxidation. J Hum Nutr Diet.

[19] Jiang R, Jacobs DR, Mayer-Davis E, Szklo M, Herrington D, Jenny NS, Kronmal R, Barr RG (2006). Nut and seed consumption and inflammatory markers in the Multi-Ethnic Study of Atherosclerosis. Am J Epidemiol.

[20] Katz DL, Davidhi A, Ma Y, Kavak Y, Bifulco L, Njike VY (2012). Effects of walnuts on endothelial function in overweight adults with visceral obesity: a randomized, controlled, crossover trial. J Am Coll Nutr.

[21] Ma Y, Njike VY, Millet J, Dutta S, Doughty K, Treu JA, Katz DL (2010). Effects of walnut consumption on endothelial function in type 2 diabetic subjects: a randomized controlled crossover trial. Diabetes Care.

[22] Casas-Agustench P, López-Uriarte P, Bulló M, Ros E, Cabré-Vila JJ, Salas-Salvadó J (2011). Effects of one serving of mixed nuts on serum lipids, insulin resistance and inflammatory markers in patients with the metabolic syndrome. Nutr Metab Cardiovasc Dis.

[23] Tapsell LC, Batterham MJ, Teuss G, Tan SY, Dalton S, Quick CJ, Gillen LJ, Charlton KE (2009). Long-term effects of increased dietary polyunsaturated fat from walnuts on metabolic parameters in type II diabetes. Eur J Clin Nutr.

[24] Mukuddem-Petersen J, Stonehouse Oosthuizen W, Jerling JC, Hanekom SM, White Z (2007). Effects of a high walnut and high cashew nut diet on selected markers of the metabolic syndrome: a controlled feeding trial. Br J Nutr.

[25] Damavandi RD, Eghtesadi S, Shidfar F, Heydari I, Foroushani AR (2013). Effects of hazelnuts consumption on fasting blood sugar and lipoproteins in patients with type 2 diabetes. J Res Med Sci.

[26] Yazdekhasti N, Mohammadifard N, Sarrafzadegan N, Mozaffarian D, Nazem M, Taheri M (2013). The relationship between nut consumption and blood pressure in an Iranian adult population: Isfahan Healthy Heart Program. Nutr Metab Cardiovasc Dis.

[27] National Health and Nutrition Examination Survey. Analytic and Reporting Guidelines. [http://www.cdc.gov/nchs/data/nhanes/nhanes_03_04/nhanes_analytic_guidelines_dec_2005.pdf] 25090154

[28] National Health and Nutrition Examination Survey. Analytic Note Regarding 2007–2010 Survey Design Changes and Combining Data Across other Survey Cycles. [http://www.cdc.gov/nchs/data/nhanes/analyticnote_2007-2010.pdf]

[29] US Department of Health & Human Services. Office of Extramural Research. [http://grants.nih.gov/grants/policy/hs/hs_policies.htm]

[30] National Health and Nutrition Examination Survey. NHANES 2009–2010 Questionnaire Data. [http://wwwn.cdc.gov/nchs/nhanes/search/datapage.aspx?Component=Questionnaire&CycleBeg nYear = 2009]

[31] Moshfegh AJ, Rhodes DG, Baer DJ, Murayi T, Clemens JC, Rumpler WV, Paul DR, Sebastian RS, Kuczynski KJ, Ingwersen LA, Staples RC, Cleveland LE (2008). The US department of agriculture automated multiple-pass method reduces bias in the collection of energy intakes. Am J Clin Nutr.

[32] Blanton CA, Moshfegh AJ, Baer DJ, The KMJ, USDA (2006). Automated multiple-pass method accurately estimates group total energy and nutrient intake. J Nutr.

[33] National Health and Nutrition Examination Survey. MEC In-Person Dietary Interviewers Procedures Manual. [http://www.cdc.gov/nchs/data/nhanes/nhanes_09_10/DietaryInterviewers_Inperson.pdf]

[34] National Health and Nutrition Examination Survey. Phone Follow-Up Dietary Interviewer Procedures Manual. [http://www.cdc.gov/nchs/data/nhanes/nhanes_09_10/Dietary_PFU_09.pdf]

[35] US Environmental Protection Agency's What We Eat in America - Food Commodity Intake Database, 2003–2008 (WWEIA-FCID 2003–08). [http://fcid.foodrisk.org]

[36] National Cancer Institute. Usual Dietary Intakes: SAS Macros for Analysis of a Single Dietary Component. [http://riskfactor.cancer.gov/diet/usualintakes/macros_single.html]

[37] National Center for Health Statistics. The NHANES Anthropometry Procedures Manual. Revised 2004. [http://www.cdc.gov/nchs/data/nhanes/nhanes_03_04/BM.pdf]

[38] National Institutes of Health. National Heart, Lung, and Blood Institute. Clinical Guidelines on the Identification, Evaluation, and Treatment of Overweight and Obesity in Adults. [http://www.nhlbi.nih.gov/guidelines/obesity/ob_gdlns.pdf]

[39] National Health and Nutrition Examination Survey. 2009 - 2010 Data Documentation, Codebook, and Frequencies. Blood Pressure (BPX_F). [http://www.cdc.gov/nchs/nhanes/nhanes2009-2010/BPX_F.htm]

[40] National Health and Nutrition Examination Survey. 2003–2004 Data Documentation, Codebook, and Frequencies. Total Cholesterol and HDL. Last revised April, 2010. [http://www.cdc.gov/nchs/nhanes/nhanes2003-2004/l13_c.htm]

[41] National Health and Nutrition Examination Survey. 2009 - 2010 Data Documentation, Codebook, and Frequencies. Cholesterol - LDL & Triglycerides (TRIGLY_F). [http://wwwn.cdc.gov/nchs/nhanes/2009-2010/TRIGLY_F.htm]

[42] National Health and Nutrition Examination Survey. 2009 - 2010 Data Documentation, Codebook, and Frequencies. Plasma Fasting Glucose and Insulin (GLU_F). [http://www.cdc.gov/nchs/nhanes/nhanes2009-2010/GLU_F.htm]

[43] Matthews DR, Hosker JP, Rudenski AS, Naylor BA, Treacher DF, Turner RC (1985). Homeostasis model assessment: insulin resistance and beta-cell function from fasting plasma glucose and insulin concentrations in man. Diabetologia.

[44] National Cholesterol Education Program. National Heart, Lung, and Blood Institute. National Institutes of Health. Detection, Evaluation, and Treatment of High Blood Cholesterol in Adults (Adult Treatment Panel III). 2002. NIH Publication No. 02–5215.

[45] O'Neil CE, Nicklas TA, Fulgoni VL (2015). Tree nut consumption is associated with better nutrient adequacy and diet quality in adults: National Health and Nutrition Examination Survey 2005–2010. Nutrients.

[46] King JC, Blumberg J, Ingwersen L, Jenab M, Tucker KL (2008). Tree nuts and peanuts as components of a healthy diet. J Nutr.

[47] United States Department of Agriculture. Agricultural Research Service. Food and Nutrient Database for Dietary Studies. [http://www.ars.usda.gov/Services/docs.htm?docid=12089]

[48] 21 CFR 101.12 - Reference amounts customarily consumed per eating occasion. [http://www.gpo.gov/fdsys/granule/CFR-2012-title21-vol2/CFR-2012-title21-vol2-sec101-12]

[49] United States Food and Drug Administration. Summary of Qualified Health Claims Subject to Enforcement Discretion. [http://www.fda.gov/food/ingredientspackaginglabeling/labelingnutrition/ucm073992.htm#nuts]

[50] Bes-Rastrollo M, Sabaté J, Gómez-Gracia E, Alonso A, Martínez JA, Martínez-González MA (2007). Nut consumption and weight gain in a Mediterranean cohort: The SUN study. Obesity (Silver Spring).

[51] Bes-Rastrollo M, Wedick NM, Martinez-Gonzalez MA, Li TY, Sampson L, Hu FB (2009). Prospective study of nut consumption, long-term weight change, and obesity risk in women. Am J Clin Nutr.

[52] Hollis J, Mattes R (2007). Effect of chronic consumption of almonds on body weight in healthy humans. Br J Nutr.

[53] Sabaté J, Cordero-Macintyre Z, Siapco G, Torabian S, Haddad E (2005). Does regular walnut consumption lead to weight gain?. Br J Nutr.

[54] Flores-Mateo G, Rojas-Rueda D, Basora J, Ros E, Salas-Salvadó J (2013). Nut intake and adiposity: meta-analysis of clinical trials. Am J Clin Nutr.

[55] Mattes RD, Dreher ML (2010). Nuts and healthy body weight maintenance mechanisms. Asia Pac J Clin Nutr.

[56] Novotny JA, Gebauer SK, Baer DJ (2012). Discrepancy between the Atwater factor predicted and empirically measured energy values of almonds in human diets. Am J Clin Nutr.

[57] Baer DJ, Gebauer SK, Novotny JA (2012). Measured energy value of pistachios in the human diet. Br J Nutr.

[58] National Institutes of Health. National Heart, Lung, and Blood Institute. What is the DASH eating plan? [http://www.nhlbi.nih.gov/health/health-topics/topics/dash]

[59] Djousse L, Rudich T, Gaziano JM (2009). Nut consumption and risk of hypertension in US male physicians. Clin Nutr.

[60] Martínez-Lapiscina EH, Pimenta AM, Beunza JJ, Bes-Rastrollo M, Martínez JA, Martínez-González MA (2010). Nut consumption and incidence of hypertension: the SUN prospective cohort. Nutr Metab Cardiovasc Dis.

[61] Casas-Agustench P, López-Uriarte P, Ros E, Bulló M, Salas-Salvadó J (2011). Nuts, hypertension and endothelial function. Nutr Metab Cardiovasc Dis.

[62] Tapsell LC, Gillen LJ, Patch CS, Batterham M, Owen A, Baré M, Kennedy M (2004). Including walnuts in a low-fat/modified-fat diet improves HDL cholesterol-to-total cholesterol ratios in patients with type 2 diabetes. Diabetes Care.

[63] Sabaté J, Fraser GE, Burke K, Knutsen SF, Bennett H, Lindsted KD (1993). Effects of walnuts on serum lipid levels and blood pressure in normal men. N Engl J Med.

[64] Sabaté J, Oda K, Ros E (2010). Nut consumption and blood lipid levels: a pooled analysis of 25 intervention trials. Arch Intern Med.

[65] Lorenzo C, Williams K, Hunt KJ, Haffner SM (2007). The national cholesterol education program adult treatment panel III, international diabetes federation, and world health organization definitions of the metabolic syndrome as predictors of incident cardiovascular disease and diabetes. Diabetes Care.

